# The Statistics of 109 Metropolitan Hospitals for 1916

**Published:** 1917-11-03

**Authors:** 


### KING EDWARD'S HOSPITAL FUND FOR LONDON.

### The Statistics of 109 Metropolitan Hospitals for 1916.

THE institutions in London owing allegiance to the Metropolitan central hospital Funds, welcoming their visiting inspectors and keeping accounts in accordance with their wishes, are stated in the Annual Statistical Report of King Edward's Hospital Fund for London to be one hundred and nine in number. Having regard to the modern meaning of the word "hospital" (which does not describe dispensaries or homes for incurables), and to the fact that two or more buildings under the same managing body have been counted as units (such as the "Dreadnought" and its branch, the Middlesex and its cancer wing, and the two hospitals of the London Lock), it may be said in round figures that the statistics of one hundred hospitals are reviewed in the report. The hospitals which are not included are not numerous and, with the exception of two or three cases, are not large; the reasons for the exclusion of them from what may be described as a federation of hospitals are various, but bear, generally speaking, no implication as to good management or respectability. In the case of the ear, nose, and throat hospitals, which are considered to be too numerous, it is probable that some pressure is being brought to bear to prepare the way for amalgamation. In another instance the Committee apparently consider that more financial prosperity will accrue to the hospital through a certain method of presenting financial statements, which is not acceptable to the Central Funds, than through falling into line with the chief hospitals of the country in the matter of uniformity of accounts.

In any case, for the purpose of arriving at useful aggregate figures, the tables relating to the 109 institutions provide very valuable data. Taken with two of the most important omissions—St. Bartholomew's Hospital and the Cancer Hospital, the list accounts for a total of 11,406 beds in average daily occupation in 1916. The total number of in-patients admitted during that year was 153,512, while 1,251,019 patients attended in the out-patient departments. Of the in-patients, 27,328 were naval and military patients treated by an understanding with the authorities, these cases occupied 25 per cent. of the total average occupied beds, which have been raised from 9,766 in 1913 to 11,406 in 1916, an increase of 1,640 beds. It follows that 1,215 beds were used by Service cases which in previous years had been at the disposal of civilians. It must, however, be borne in mind that a large part of the male population is serving with the Forces, and consequently many soldiers are receiving a privilege that in normal times they would be granted as civilians.

Still including the two hospitals last quoted, the total ordinary expenditure at these hospitals, including maintenance, administration, rent, rates and taxes, but excluding capital expenditure, interest on borrowed money, and contributions to convalescent homes and country branches, amounted in 1916 to £1,513,834. It is claimed in the report that as the receipts from the authorities for the maintenance of sailors and soldiers amounted to £218,087, only £1,295,747 had to be met out of the normal sources of hospital revenue. We do not like this confusion of the two sides of the income and expenditure account. The financial argument is not sound, because it surmises that the hospitals would, if there had been no wounded, have filled the beds with free patients. This is quite too large an assumption; moreover the possibility is overlooked that a hospital may have given up some of the "normal sources of income" in order to take Service patients. This, to our knowledge, is the case in one instance where a large ward receiving patients at £1 11s. 6d. a week each has been devoted to the use of soldiers at £1 8s. a week each.

It is not surprising to learn that in 1916 the increase of expenditure "to be borne by the hospitals" (the perplexing financial argument quoted above qualifies the figure) was £104,540, or 8.77 per cent. The aggregate increase was £171,707, or 12.79 per cent.

